# Combining neurobiological markers and a sociodemographic risk score to predict adolescent depression – An IDEA RiSCo prospective cohort study

**DOI:** 10.1038/s41380-026-03481-y

**Published:** 2026-03-02

**Authors:** Zuzanna Zajkowska, Naghmeh Nikkheslat, Pedro H. Manfro, Laila Souza, Fernanda Rohrsetzer, Anna Viduani, Rivka Pereira, Jader Piccin, Leehyun Yoon, Valentina Zonca, Helen L. Fisher, Johnna R. Swartz, Brandon A. Kohrt, Christian Kieling, Valeria Mondelli

**Affiliations:** 1 King’s College London, Department of Psychological Medicine, Institute of Psychiatry, Psychology & Neuroscience, London, UK; 2 Department of Psychiatry, Universidade Federal do Rio Grande do Sul, Child & Adolescent Psychiatry Division, Hospital de Clínicas de Porto Alegre, Porto Alegre, Brazil; 3 Department of Psychology, University of Texas at Dallas, Richardson, USA; 4 Department of Pharmacological and Biomolecular Sciences, University of Milan, Milan, Italy; 5 King’s College London, Social, Genetic & Developmental Psychiatry Centre, Institute of Psychiatry, Psychology & Neuroscience, London, UK; 6 ESRC Centre for Society and Mental Health, King’s College London, London, UK; 7 Department of Human Ecology, University of California, Davis, USA; 8 Department of Psychiatry and Behavioral Health, The George Washington University, 2120 L St NW, Ste 600, Washington, DC 20037 USA; 9 Instituto de Pesquisa, Hospital Moinhos de Vento, Porto Alegre, RS Brazil; 10 National Institute for Health Research (NIHR) Mental Health Biomedical Research Centre at South London and Maudsley NHS Foundation Trust and King’s College London, London, UK

**Keywords:** Predictive markers, Neuroscience

## Abstract

The prevention of adolescent depression is a critical global challenge, hindered by the difficulty of identifying those at increased risk. We previously developed a sociodemographic-based risk score (IDEA-RS), which has proven to successfully predict depression onset in adolescents across five continents. This study aims to 1) improve the predictive accuracy by integrating biological and sociodemographic factors, and 2) generate a biological risk score, by combining biological information, to complement IDEA-RS. Derived from screening over 7000 adolescents aged 14–16 years old in Brazil, we recruited 100 adolescents representing the extremes of the sociodemographic risk score and including 50 adolescents at low-risk and 50 at high-risk of developing depression according to IDEA-RS. Baseline blood samples and fMRI scan were performed to measure selected biological markers: cytokines, kynurenine pathway metabolites and amygdala reactivity to negative stimuli. Adolescents were followed up over three years to identify those who would transition to develop depression. The primary outcome of depression incidence was assessed using the Schedule for Affective Disorders and Schizophrenia for School-Age Children-Present and Lifetime Version (K-SADS-PL). A composite biological risk score (IDEA-BIO-RS) was created incorporating the above biomarkers. Combining biological factors with IDEA-RS significantly improved the accuracy of predicting depression onset compared with IDEA-RS alone. Among adolescents classified as high-risk by both IDEA-RS and IDEA-BIO-RS, 44% developed depression over three years, while none of those classified as low-risk by both scores developed depression. This combined approach shows promise as a screening tool to identify adolescents at risk of developing depression and aid in prevention efforts.

## Introduction

In the last decade, the rates of depression have increased in adolescents posing a significant burden on individual and societal levels globally [1, 2]. Developing effective early prevention strategies is crucial, but identifying at-risk adolescents remains challenging due to a lack of integrated approaches that consider socioenvironmental and biological factors, especially in low- and middle-income countries (LMICs) where most adolescents live and where limited research exists [3–8].

The increasing evidence highlights the interaction between socioenvironmental and neurobiological factors in depression [9, 10]. We have previously identified the main environmental and sociodemographic risk factors associated with increased risk of developing adolescent depression [11]; and demonstrated that a composite risk score (IDEA-RS) based only on sociodemographic variables has shown predictive accuracy of 66–83% across five continents [12**–**16]. Here we will integrate neurobiological markers to understand if we can improve the identification of increased risk of adolescent depression. In particular, we will focus on inflammatory markers, kynurenine pathway markers of neuroprotective/neurotoxic balance, and amygdala activity to negative stimuli since these are among the most studied biomarkers associated with depression and previously linked to increased risk of adolescent depression.

Increased inflammation is among the main biological mechanisms recently suggested to play a role in the development of depression. Among the most consistently studied inflammatory markers, interleukin (IL)-6 and tumour necrosis factor (TNF)-α are the ones to be more frequently found elevated in adolescents with depression as well as prior to its onset, suggesting they might be a helpful tool in identifying at-risk individuals [8, 17–21]. Along with IL-6 and TNF- α, the less commonly investigated cytokines IL-2 and IL-12p70 have also been shown to be compelling candidates as risk factors for adolescent depression as both have been found to be elevated in adolescent depression [20], with IL-2 being particularly relevant in adolescents studied in LMICs contexts [19]. One of the mechanisms through which inflammation may contribute to the development of depression is by disrupting the balance between neuroprotective and neurotoxic brain metabolites of the kynurenine pathway, increasing neurotoxic metabolites like quinolinic acid and reducing neuroprotective ones like kynurenic acid [22, 23]. Considering the simultaneous and competing effects of these metabolites, the ratio of kynurenic and quinolinic acid (KA/QA) represents an optimal measure of the balance of neuroprotection over neurotoxicity [24]. Although evidence in adolescence is limited, our recent study by Nikkheslat et al. [25] is in line with the adult literature, demonstrating a lower KA/QA ratio in adolescent females who were either at high risk for depression or had MDD when compared with those at low risk for depression. As such, we will focus on IL-6, TNF- α, IL-2 and IL-12p70 as the main inflammatory markers and on KA/QA ratio as the marker of the balance of neuroprotection over neurotoxicity.

Another important neurobiological marker to consider is the measure of amygdala activity in response to negative emotional stimuli, as an increase in this activity is a well-established marker of depression, observed in both adults and adolescents [26]. Studies show that children and adolescents with a family history of depression or other risk factors show greater amygdala activity to negative emotional stimuli, such as sad and fearful faces, than their low-risk peers [27**–**30]. Research in young adults indicates that heightened amygdala activity to negative emotional faces predicts subsequent increased internalizing symptoms in those who experience high levels of stressful life events, suggesting it could be a potential biomarker for adolescent depression risk [31].

We have recently demonstrated in a prospective cohort study (IDEA-RiSCo) that adolescents stratified as high-risk according to our composite risk score (IDEA-RS) were at more than 2.5 times greater risk for developing depression at 3-year follow-up compared with those classified as low-risk [32]. In the present study we present analyses on whether the accuracy of our prediction of adolescent depression risk improves when integrating data on neurobiological risk markers.

This study aimed to investigate whether neurobiological markers associated with depression (i.e. inflammatory cytokines, kynurenine pathway markers of reduced neuroprotectivity, and amygdala activity to negative emotional stimuli) improve the accuracy of the IDEA-RS in predicting depression onset in at-risk adolescents. The second aim was to develop a composite biological risk score (IDEA-BIO-RS) and to test its predictive ability for adolescent depression independently of and together with the IDEA-RS.

## Methods and materials

### Ethics approval

This study was approved by the Brazilian National Ethics in Research Commission (CAAE 50473015.9.0000.5327), the Ethics Committee at King’s College London for Psychiatry, Nursing & Midwifery for secondary data analysis for biological measures (LRS-17/18-8327), and the Institutional Review Board at the University of California, Davis for the analysis of the functional MRI data (1218177-4). All adolescents provided their written informed assent, and their caregivers provided written informed consent before taking part in the study.

### The main outcome

The main outcome was development of depression within a 3-year period at any time since baseline. At both baseline and the 3-year follow-up assessment, presence/absence of depression was assessed by board-certified child and adolescent psychiatrists using the Brazilian Portuguese translation of the depression module (including dysthymia) of the Schedule for Affective Disorders and Schizophrenia for School-Age Children-Present and Lifetime Version (K-SADS-PL) [33]. Whereas the baseline diagnostic interviews were conducted in person with both the participant and their primary caregiver, at 3-year follow-up all diagnostic interviews were performed online via telemedicine with information obtained exclusively from the participants, retrospectively.

### Recruitment procedure and group ascertainment

This study is part of the wider IDEA-RiSCo study, investigating risk factors for depression in adolescence [11, 34, 35].

We used the IDEA risk score (IDEA-RS), which was previously developed to estimate the future probability of a diagnosis of major depressive disorder in adolescence [16], to screen 7720 adolescents across public schools in Porto Alegre, Brazil. Out of these, we selected and included in the study 100 adolescents stratified into two distinct risk categories for developing depression, based on the IDEA-RS, representing the extremes of the sociodemographic risk. The two groups included: 1) one group of adolescents at low-risk of developing depression scoring equal to or below the 20^th^ percentile of the IDEA-RS (n = 50) and a Patient Health Questionnaire – Adolescent Version (PHQ-A) score lower than 6, and 2) one group of adolescents at high-risk of developing depression scoring equal to or above the 90^th^ percentile of the IDEA-RS (n = 50), also with a PHQ-A score lower than 6 [36]. Participants were selected to have an even sex distribution within each group (25 females and 25 males for each group). Please see Table S1 in Supplementary Material for details of the IDEA-RS variables and their distribution between the two groups.

Participants were followed up for 3 years - at 1 year (retention rate 96%), 2 years (retention rate 94%), and 3 years (retention rate 88%). Out of 88 participants who completed the follow-up at 3 years (including n = 43 from the low-risk group and n = 45 from the high-risk group), a total of 19 adolescents developed depression at some point during the 3-year follow-up period. Of the 19 adolescents who developed depression, 14 were at high-risk and 5 were at low-risk for depression according to the IDEA-RS at baseline (see Table 1).

**Table 1:** Sample demographics.

Characteristics	Low risk (n = 50) Mean (SD)^a^	High risk (n = 50) Mean (SD) ^a^	Group Comparisons & Post-hoc Analysis
Girls (n)	25	25	-
Age (years)	15.4 (0.81)	15.8 (0.83)	U = 1604, z = 2.440, p = 0.015
Anxiety symptoms (SCAS-C)	23.02 (11.03)	25.5 (11.3)	U = 1448. z = 1.367, p = 0.172
Transition to MDD FU	5	14	-
Girls (n) FU	23	21	-
Boys (n) FU	22	24	-
Age FU (years)	17.8 (0.84)	18.4 (0.96)	U = 1122, z = 2.522, p = 0.012

*SD* standard deviation, *MDD* major depressive disorder, *FU* follow-up, *SCAS-C* spence children’s anxiety scale child version.

^a^Unless noted as (n).

At the time of recruitment, adolescents were aged 14–16, and at the last follow-up 17–19 (see Table 1). Baseline exclusion criteria included any inflammatory condition or active infection, use of psychotropic medication over the last 30 days, use of anti-inflammatory medication over the last 14 days, brain malformations, epilepsy, left-handedness, IQ < 70, and presence of other psychiatric conditions such as autism spectrum disorder, bipolar disorder, eating disorders, post-traumatic stress disorder, schizophrenia, or substance use disorders. For more details, please see our previous publication [11]. Baseline assessments included blood collection for cytokine and kynurenine metabolites measures and MRI brain scans for neuroimaging.

### Cytokine analysis

We measured serum cytokine levels using Meso Scale Discovery (MSD) electrochemiluminescence V-PLEX assay. Based on data from our and others’ previous studies, we included in this study only the main cytokines previously found to be significantly associated with adult or adolescent depression [8, 17–21]; these included: IL-2, IL-6, IL-12p70 and TNF-α (pg/mL). Serum samples were diluted 2-fold and measured in duplicate. The concentration of cytokines was determined via electrochemiluminescent labels whilst the plate was inserted into the MSD instrument (MESO QUICKPLEX SQ 120). The inter-assay coefficient of variations was <10%. The results were analysed using MSD DISCOVERY WORKBENCH analysis software. For more details, please see our previous publication [8].

### Kynurenine pathway analysis

Kynurenine pathway metabolites were analysed from plasma samples using targeted ultrahigh-performance liquid chromatography tandem mass spectrometry with electrospray ionization (UHPLC-ESI-MS/MS) method. For the purpose of this study, we focused only on the ratio between kynurenine acid and quinolinic acid (KA/QA), which is the measure of the balance between neuroprotective and neurotoxic metabolites from the kynurenine pathway and previously reported to be altered in MDD [24]. Further details on the method used can be found in our previous publication [25].

### Amygdala activity to negative stimuli

The functional MRI (fMRI) data were acquired from a 3 T Ingenia scanner (Koninklijke Philips N.V., Amsterdam, The Netherlands), software version 5.3.1 at Hospital de Clinicas de Porto Alegre, Universidade Federal do Rio Grande do Sul, Porto Alegre, Brazil. As reported in Yoon et al. [37], 41 low-risk and 41 high-risk adolescents had fMRI data meeting all quality control criteria for the face task at baseline [37]. For the current analysis, we selected measures from the fMRI analysis where we assessed amygdala activity to negative emotional stimuli, which included measures of amygdala activity to fearful, sad and angry faces. Measures of amygdala activity were extracted for each condition (e.g., fearful face matching vs. shape matching control condition) for the left and right amygdala using an anatomically-defined region of interest based on the Automated Anatomical Labeling Atlas [38]. Measures of amygdala activity for the left and right amygdala for each condition were averaged so that there was one measure of amygdala activity for each emotional face expression. During fMRI scanning, participants completed two tasks including a face matching task in which they were asked to identify which of two faces matched a target face via button press. Full details on the methods of acquisition, pre-processing, quality control, and first-level and group-level analysis can be found in our previous publication [37].

### Development of the IDEA biological risk score (IDEA-BIO-RS)

A biological risk score (IDEA-BIO-RS) including all eight biological variables (i.e. IL-2, IL-6, IL-12p70, TNF-α, KA/QA, amygdala activity to (i) fearful, (ii) sad, and (iii) angry faces) was developed by dichotomising each biological variable based on their median within the overall sample. Based on the existing literature demonstrating increased levels in depression of all the above biological factors except KA/QA ratio, we considered the scores equal to and above the median to indicate high-risk for depression for all variables except KA/QA ratio [39]. For KA/QA this was coded in reverse (e.g. scores equal to and above the median were considered to indicate low-risk for depression) as higher KA/QA is indicative of higher neuroprotection vs neurotoxicity. We summed those scores to create a biological risk score (IDEA-BIO-RS) ranging from 0 to 8, where 0 indicated the lowest and 8 the highest risk for developing depression. Based on the median score, we dichotomized the IDEA-BIO-RS into low-risk ( < 4) and high-risk (>=4) for future onset of adolescent depression. Because not all participants met quality control criteria for the fMRI data, only 73 participants had data available for all eight biological variables, as well as follow-up data on depression diagnoses, and were included in the calculation of the IDEA-BIO-RS. Of the 15 participants who were excluded because of not meeting quality control criteria for MRI, n = 2 developed depression at follow-up. Descriptive values of the inflammatory markers for the low- and high-risk IDEA-BIO-RS groups are presented in Table S2 (Supplementary Material).

### Data analysis

Statistical analysis was performed using IBM SPSS statistical software version 29. To analyse the predictive accuracy of IDEA-RS and biomarkers in transition to depression, we used binomial logistic regression. First, we performed separate regressions using IDEA-RS together with 1) cytokines, 2) kynurenine metabolite ratio, and 3) brain functional imaging included as continuous variables. Then we combined the IDEA-RS and all the biomarkers in one regression model followed by the ROC curve to determine the classification performance of the combined model. For regression models, we used either raw or natural log transformed data based on the best model fit.

In order to test incidence rates of transition to depression in low-risk and high-risk groups according to the IDEA-BIO-RS, we performed a Chi-square test of independence. To test incidence rates of transition to depression in low-risk and high-risk groups according to biological and sociodemographic risk, we combined the IDEA-BIO-RS and IDEA-RS into one categorical variable with four levels: low-risk IDEA-RS and low-risk IDEA-BIO-RS (LL group), low-risk IDEA-RS and high-risk IDEA-BIO-RS (LH group), high-risk IDEA-RS and low-risk IDEA-BIO-RS (HL group), high-risk IDEA-RS and high-risk IDEA-BIO-RS (HH group). Using this combined risk score, we performed Chi-square tests of independence to test incidence rates for each of the four categories and transition to depression at follow-up.

### Power calculation

The sample size was initially determined based on the original study protocol, which focused on cross-sectional analyses [11]. For the longitudinal analyses presented in the current paper, focusing on 100 participants from the LR and HR groups, we assumed an 80% retention rate at the 3-year follow-up, based on retention rates of approximately 85% observed in the previous Pelotas cohort study [40]. This resulted in an expected follow-up sample of approximately 80 participants. Power calculations conducted using G*Power software indicated that a sample of 80 participants would provide 85% power to detect a medium effect size (odds ratio = 2.5) in logistic regression analyses, with a two-tailed significance level of α = 0.05.

## Results

### IDEA-RS with baseline cytokines as predictors of transition to depression within the 3-year follow-up

When testing the predictive performance of cytokines (IL-2, IL-6, IL-12p70, and TNF-α) together with the IDEA-RS on transition to depression within the 3-year follow-up, we found that this logistic regression model demonstrated improved prediction of transition to depression than using the IDEA-RS on its own by 19.1%, explaining 28.1% (Nagelkerke R^2^) of the variance and correctly classifying 81.8% of participants (X^2^ (5) = 17.67, p = 0.003) (see Table 2).

**Table 2:** IDEA-RS with baseline cytokines as predictors of transition to depression within the 3-year follow-up.

IDEA-RS and Cytokines (pg/mL) (n = 88)	Coefficient Exp (B)	95% CI	p value
IDEA-RS	1.108	0.989, 1.243	0.078
IL-2	1.521	0.806, 2.868	0.195
IL-6	2.553	0.810, 8.047	0.110
IL-12p70	1.762	0.797, 3.169	0.059
TNF-α	0.118	0.008, 1.701	0.117

Overall model statistics: X^2^ (5) = 17.67, p = 0.003.

*IDEA-RS* IDEA risk score, *IL* interleukin, *TNF-α* tumour necrosis factor alpha, *CI* confidence interval.

### IDEA-RS with baseline kynurenic acid/quinolinic acid ratio as predictors of transition to depression within the 3-year follow-up

When testing the predictive performance of KA/QA ratio together with the IDEA-RS on the transition to depression within the 3-year follow-up, we found that this logistic regression model improved our prediction of transition to depression by 6.9% explaining 15.9% (Nagelkerke R^2^) of the variance, correctly classifying 78.4% of participants (X^2^ (2) = 9.53, p = 0.009; Exp (B) = 0.829, 95% CI 0.657, 1.047, p = 0.115).

### IDEA-RS with baseline amygdala activity to negative stimuli as predictors of transition to depression within the 3-year follow-up

The sample size with neuroimaging data was slightly smaller (n = 73) than the one with blood-based biomarkers, as not every participant passed quality control criteria for fMRI. When testing the predictive performance of amygdala activity to fearful, sad, and angry faces together with the IDEA-RS on the transition to depression within the 3-year follow-up, we found that this logistic regression model explained 17.4% (Nagelkerke R^2^) of the variance, correctly classifying 78.1% of participants, although the model was at a trend level for statistical significance (X^2^ (4) = 8.91, p = 0.06) (see Table 3).

**Table 3:** IDEA-RS with baseline amygdala activity to negative stimuli as predictors of transition to depression within the 3-year follow-up.

IDEA-RS and Amygdala activity (n = 73)	Coefficient Exp (B)	95% CI	p value
IDEA-RS	1.163	1.027, 1.318	0.017
fearful faces	3.311	0.585, 18.732	0.176
sad faces	0.521	0.055, 4.914	0.569
angry faces	0.804	0.126, 5.151	0.818

Overall model statistics: X^2^ (4) = 8.91, p = 0.06.

*IDEA-RS* IDEA risk score, *CI* confidence interval.

### IDEA-RS with all the biological risk factors combined as predictors of transition to depression within the 3-year follow-up

We then tested whether adding all eight biological risk factors together with the IDEA-RS into a single logistic regression model would improve our prediction of transition to depression within the 3-year follow-up. We found that after adding all the biological risk factors to the IDEA-RS, our model improved by 40.8% and explained 49.8% (Nagelkerke R^2^) of the variance, correctly classifying 82.2% of participants (X^2^ (9) = 29.24, p < 0.001) (see Table 4).

**Table 4:** IDEA-RS with all the biological risk factors combined as predictors of transition to depression within the 3-year follow-up.

IDEA-RS and all biological risk factors (n = 73)	Coefficient Exp (B)	95% CI	p value
IDEA-RS	1.144	0.976, 1.340	0.098
IL-2	1.952	0.847, 4.499	0.116
IL-6	4.860	1.041, 22.703	0.044
IL-12p70	1.678	0.821, 3.428	0.156
TNF-α	0.034	0.001, 0.915	0.044
KA:QA	0.790	0.579, 1.078	0.137
fearful faces	6.330	0.639, 62.704	0.115
sad faces	0.344	0.013, 9.336	0.526
angry faces	0.983	0.091, 10.652	0.989

Overall model statistics: X^2^ (9) = 29.24, p < 0.001.

*IDEA-RS* IDEA risk score, *IL* interleukin, *TNF-α* tumour necrosis factor alpha;, KA:QA: kynurenic acid to quinolinic acid ratio, *CI* confidence interval.

**Fig. 1:**
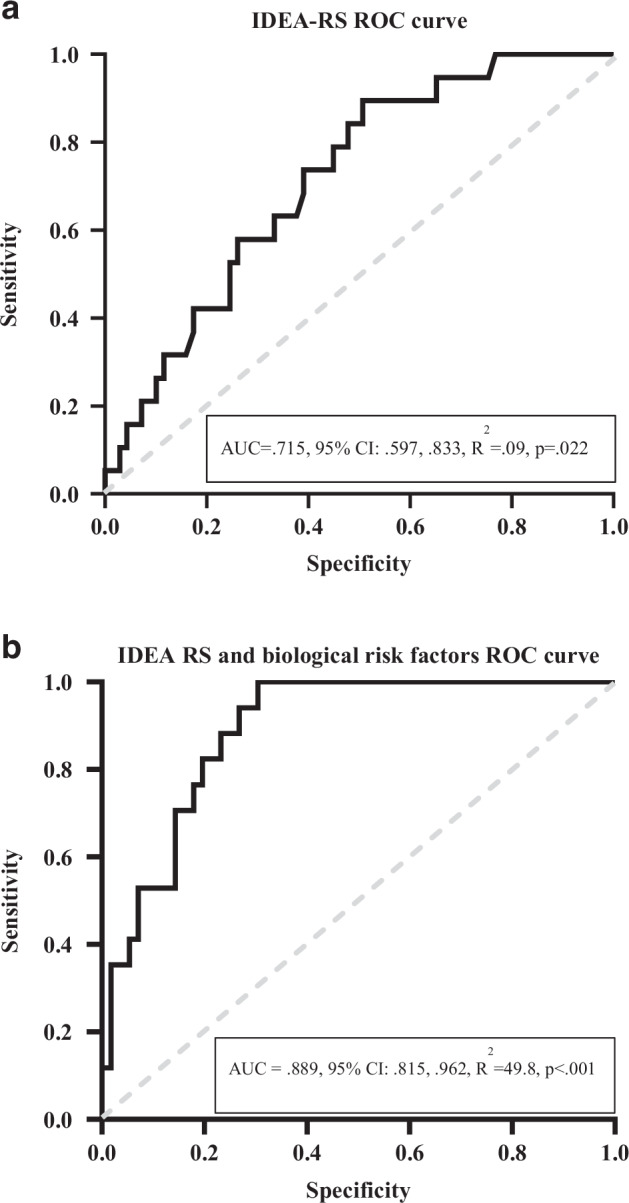
**A** Binomial logistic regression – IDEA-risk score (IDEA-RS) as a predictor of the transition to depression within the 3-year follow-up. **B** Binomial logistic regression – IDEA-risk score (IDEA-RS) and biological risk factors combined (IL-12p70, IL-2, IL-6, TNF-α, KA/QA ratio, and amygdala reactivity to sad, fearful and angry faces) as predictors of the transition to depression within the 3-year follow-up.

We then tested the ROC curve for the models. For reference from our previous findings [32], the ROC curve including only the IDEA-RS on its own as a predictor of transition showed that the model was able to discriminate adolescents who transitioned to depression with moderate accuracy with an area under the curve (AUC) of 0.715 (95% CI 0.597, 0.833) (see Fig. 1A). The new ROC curve from the current study including all biological markers together with the IDEA-RS showed that the model was able to discriminate adolescents who transitioned to depression with substantial accuracy with an AUC = 0.889 (95% CI 0.815, 0.962) (see Fig. 1B).

### Incidence rates of transition to depression within the 3-year follow-up based on the IDEA-BIO-RS and IDEA-RS

**Fig. 2:**
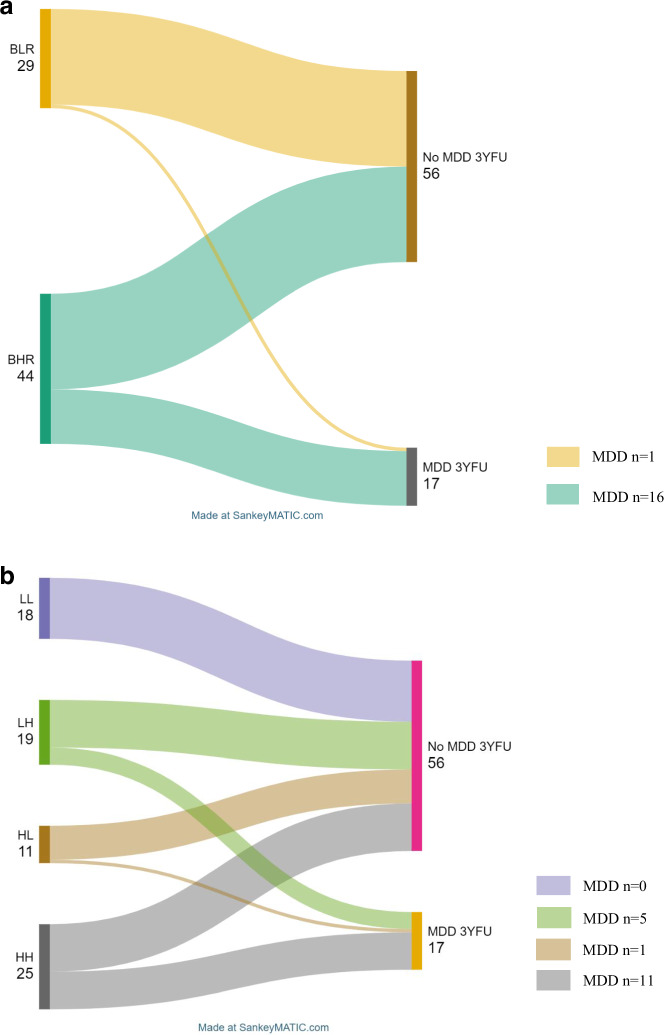
**A** Chi-square test of independence – incidence rates of transition to depression within the 3-year follow-up in adolescents at low (BLR) and high (BHR) biological risk for depression at baseline. **B** Chi-square test of independence – incidence rates of transition to depression within the 3-year follow-up in adolescents grouped into 4 categories of risk according to baseline IDEA risk score (IDEA-RS) and biological risk score (IDEA-BIO-RS): low risk IDEA-RS and low risk BIO-RS (LL group), low risk IDEA-RS and high-risk BIO-RS (LH group), high risk IDEA-RS and low BIO-RS (HL group), high risk IDEA-RS and high risk BIO-RS (HH group).

In our next analyses we investigated incident rates of transition to depression within the 3-year follow-up in adolescents at baseline low vs high risk according to the IDEA-BIO-RS. Chi-square analysis showed that 16 (36.4%) adolescents at biological high risk (BHR) for depression at baseline developed depression at the follow-up whilst 28 (63.6%) did not, whereas in the biological low risk (BLR) group only 1 (3.4%) ended up developing depression and 28 (96.6%) did not (x^2^(1) = 10.601, p = 0.001) (see Fig. 2A).

When we looked at IDEA-BIO-RS and IDEA-RS combined and categorised them into 4 risk groups (LL, LH, HL, and HH), we found that amongst 18 adolescents in the LL group, none of them transitioned to depression at the follow-up, in the LH group 5 adolescents (26.3%) transitioned to depression whereas 14 did not, in the HL group 1 adolescent (9.1%) transitioned to depression and 10 did not, and lastly in the HH group 11 adolescents (44.0%) transitioned to depression at the follow-up and 14 did not (x^2^(3) = 12.806, p = 0.005) (see Fig. 2B).

## Discussion

This study shows that combining neurobiological markers with sociodemographic risk factors significantly improves prediction of adolescent depression compared to using sociodemographic factors alone. Specifically, adding biomarkers such as inflammatory cytokines, kynurenine pathway markers, and amygdala activity to the IDEA-RS score enhanced prediction accuracy within a 3-year follow-up. Combining the IDEA-RS with biological risk factors improved our ability to identify adolescents at higher risk of depression, showing substantial discriminative ability. Notably, 44% of adolescents classified as high-risk on both biological (IDEA-BIO-RS) and sociodemographic (IDEA-RS) factors developed depression, compared to 26.3 and 9.1% for those classified as high-risk on either factor alone. Importantly, none of the adolescents classified as low-risk on both biological (IDEA BIO-RS) and sociodemographic (IDEA-RS) factors developed depression during the follow-up period.

Our findings showing immune and neuroimaging markers as predictors of adolescent depression are consistent with previous literature [21,31, 41]. While most studies focus on single biological markers for depression, our study shifted to a composite risk score approach, combining biomarkers known to contribute to depression [17, 21, 42]. This is particularly relevant in the field of psychiatry where single biomarker-based approaches are unlikely to achieve clinically relevant predictive ability considering the heterogeneity and complex etiopathogenesis of mental health disorders [43]. Recent studies in the field of mental health have tried to integrate multiple biomarkers using mainly machine learning models [44, 45]. However, predictive algorithms from these models can be complex and hard to interpret, limiting replicability and clinical applicability. In contrast, a simple biological risk score, like the one developed in this study, offers easier replication and potential clinical use.

Examining biomarkers linked to depression’s etiopathogenesis can clarify its biological mechanisms and aid in developing effective preventive interventions. Our study focused on three biomarker sets: 1) immune markers, 2) neuroprotection/neurotoxicity markers, and 3) neural markers of emotional processing, which have shown interactions in previous research

[46, 47]. For example, one of the mechanisms through which immune activation can contribute to the development of depression is the regulation of the kynurenine pathway and the balance between neuroprotective and neurotoxic metabolites [24]. Similarly, previous studies have shown a link between increased peripheral inflammation and higher activity of the amygdala in response to emotional stimuli [48, 49]. A recent review proposed a neuroimmune network model of depression in adolescents, suggesting a bi-directional link between immune activation and brain function, including heightened amygdala activity to negative stimuli [46].

Interestingly, our data show that some biomarkers (i.e. TNF-alpha, amygdala reactivity to sad and angry faces) did not follow the expected direction of association, based on previous literature. Combining these biomarkers significantly improved depression prediction, emphasizing the value of composite risk scores over individual markers. While it remains unclear whether these biomarkers represent distinct or overlapping pathways, the increased predictive ability suggests they may contribute independently or reflect different stages of the same biological pathway. Grouping cytokines, rather than testing each individually, allows for a broader assessment of interconnected immune pathways, offering deeper insights into depression. As we demonstrate in the heatmap (Figure S1, Supplementary Material), in the HH group, higher levels of TNF-α, amygdala activity to sad faces and KA/QA ratio were observed in males, whereas lower levels were found in females. This sex-related difference may partly explain the deviation from expected associations and underscores the importance of accounting for sex-specific biological variability when refining predictive models and maximising scalability in LMICs. This finding aligns with our previous findings from the same cohort, which demonstrated sex-specific differences in cytokine and kynurenine pathway metabolites in relation to depression [8, 25]. We also found that increased IL-2 levels were related to better-quality relationships with the father and between parents, while higher IL-12p70 levels were specifically associated with a better paternal relationship. These novel and noteworthy associations merit further exploration, particularly within LMIC contexts, where research on psychosocial and immunological factors remains limited. Recent evidence indicates that depression phenotypes and their biological correlates may differ across sociocultural and economic contexts. Studies from certain LMIC settings, for example, have reported higher prevalence of inflammation, which might partly explain the predictive performance observed in our sample [50]. Another major novel aspect of this study is integrating sociodemographic and biological risk factors into a multidimensional risk score such as the Framingham Risk Score for coronary heart disease. It collates information from six variables, (demographic and biological), and has proven effective in predicting the risk of developing coronary heart disease. Unlike most studies on adolescent depression, which focus on a single biological factor, this study combines multiple types of risk for more comprehensive prediction [9].

In our recent publication, we demonstrated that high-risk adolescents identified by the IDEA-RS were nearly three times more likely to develop depression over a 3-year follow-up compared to low-risk peers [32]. This study shows that adding biological risk factors to sociodemographic data improves depression prediction accuracy, achieving a better model performance with all biological factors included. To our knowledge, this is the first study to propose a composite biological risk score for identifying future depression risk in adolescents. Combining IDEA-RS and IDEA-BIO-RS could help develop a screening tool for early identification and prevention of depression in at-risk adolescents.

Although the sample size was relatively small, our baseline stratification approach for sociodemographic risk enabled the inclusion of two extreme risk populations, allowing for deep biological phenotyping not feasible in a larger cohort. A potential drawback is that our sample includes only individuals at sociodemographic risk extremes (using IDEA-RS), likely enhancing the model’s ability to predict depression better than a general population sample. However, our sample was not stratified for biological factors and 7 out of 8 biomarkers were not associated with increased sociodemographic risk as previously reported [8, 25, 37]; therefore, the stratification for sociodemographic risk seems not to have affected the distribution of the biological variables. However, larger and more diverse population studies are needed to validate our findings and confirm the potential of IDEA-BIO-RS.

Another potential limitation was that the development of depression since baseline was assessed retrospectively. However, this was mitigated by the fact that PHQ-A scores collected prospectively at the 1- and 2-year follow-ups corroborated the retrospective K-SADS findings, providing convergent validity for the retrospective assessment. In addition, a sensitivity analysis using a PHQ-A-based algorithm to identify depressive episodes during follow-up intervals yielded consistent results, confirming that the elevated risk of depression among HR adolescents was not dependent on the method of ascertainment. These findings suggest that, while retrospective assessment represents a methodological limitation, it did not substantially bias the study outcomes [32]. Although one of the main strengths of the study is the large number of biomarkers in the same cohort, we acknowledge that other biomarkers (e.g. telomere length) could potentially be of interest in the prediction modelling; the choice was partly driven by biological markers with the strongest prior evidence of their role in the development of adolescent depression. Larger cohort studies are needed to confirm the generalizability of these biomarkers and their relevance to both sexes, given prior findings on sex differences in inflammatory markers in this cohort [8]. Indeed, a limitation of this study was that the sample size was too small to permit us to examine effects separately by sex.

Building on our findings, it is important to consider the practical implications of incorporating biological risk factors into assessment procedures. While such measures may enhance predictive precision, they also introduce additional complexity and cost, and their feasibility likely varies across clinical and community settings. A stepwise screening approach could offer a pragmatic solution, whereby low-cost, accessible indicators are used initially and more resource-intensive biological assessments are reserved for individuals at higher risk. Blood-based biomarkers appear relatively feasible given their established clinical utility and modest cost. In contrast, neuroimaging measures remain less accessible, particularly in resource-limited settings, constraining their scalability. Replication in larger and more diverse cohorts is needed to establish whether the observed improvements in classification accuracy justify the additional financial and logistical demands associated with MRI-based assessments.

In conclusion, adding biological markers improves the prediction of adolescent depression beyond sociodemographic risk, with combined markers enhancing accuracy. Our biological risk score identified high-risk adolescents missed by the sociodemographic score. Integrating IDEA-RS and IDEA-BIO-RS marks a step toward developing a multi-step screening tool to identify at-risk adolescents and accelerate early prevention strategies.

## Supplementary information

Supplementary Material
