# Supplementary material for: Combining neurobiological markers and a sociodemographic risk score to predict adolescent depression – An IDEA RiSCo prospective cohort study
Source: Mol Psychiatry. 2026 Mar 2;31(6):3516–23. doi: 10.1038/s41380-026-03481-y (PMC13190231; doi:10.1038/s41380-026-03481-y)
Supplement: Supplementary file 1 — Supplementary Material [file 41380_2026_3481_MOESM1_ESM.docx]

**Supplementary Material**

**Zajkowska et al. Combining neurobiological markers and a sociodemographic risk score to predict adolescent depression – An IDEA RiSCo prospective cohort study**

Table S1. The distribution of 11 sociodemographic characteristics that determine the IDEA-RS across low and high-risk groups.

| **Sociodemographic characteristics** | **Low risk (n=50)**  **N (%)** | **High risk (n=50)**  **N (%)** |
| --- | --- | --- |
| Sex, female | 25 (50%) | 25 (50%) |
| Skin colour, non-white | 22 (44%) | 26 (52%) |
| Not meeting friends | 1 (2%) | 10 (20%) |
| School failure | 0 | 29 (58%) |
| Ran away from home | 1 (2%) | 3 (6%) |
| Any drug use | 29 (58%) | 44 (88%) |
| Fights | 0 | 20 (40%) |
| Relationship with father (mean, SD) | 4.52 (0.79) | 2.48 (1.22) |
| Relationship with mother (mean, SD) | 4.78 (0.55) | 3.92 (1.01) |
| Relationship between parents (mean, SD) | 4.18 (1.08) | 2.38 (1.23) |
| Childhood maltreatment  None  Probable  Severe | 50 (100%)  0  0 | 1 (2%)  12 (24%)  37 (74%) |

IDEA-RS: IDEA risk score; SD: standard deviation

Table S2. Descriptive values of the inflammatory markers in the low and high-risk IDEA-BIO-RS groups.

| Cytokine (pg/mL) | Low Risk IDEA-BIO-RS  Mean (SD) | High Risk IDEA-BIO-RS  Mean (SD) |
| --- | --- | --- |
| IL-2 | 0.15 (0.23) | 0.19 (0.14) |
| IL-6 | 0.33 (0.13) | 0.52 (0.26) |
| IL-12p70 | 0.1 (0.12) | 0.14 (0.14) |
| TNF-α | 2.6 (0.59) | 3.22 (0.81) |

IDEA-BIO-RS: IDEA biological risk score; SD: standard deviation; IL: interleukin; TNF-α: tumour necrosis factor alpha

Figure S1. Heatmap - Distribution of the sociodemographic and biological risk factors in the HH group.


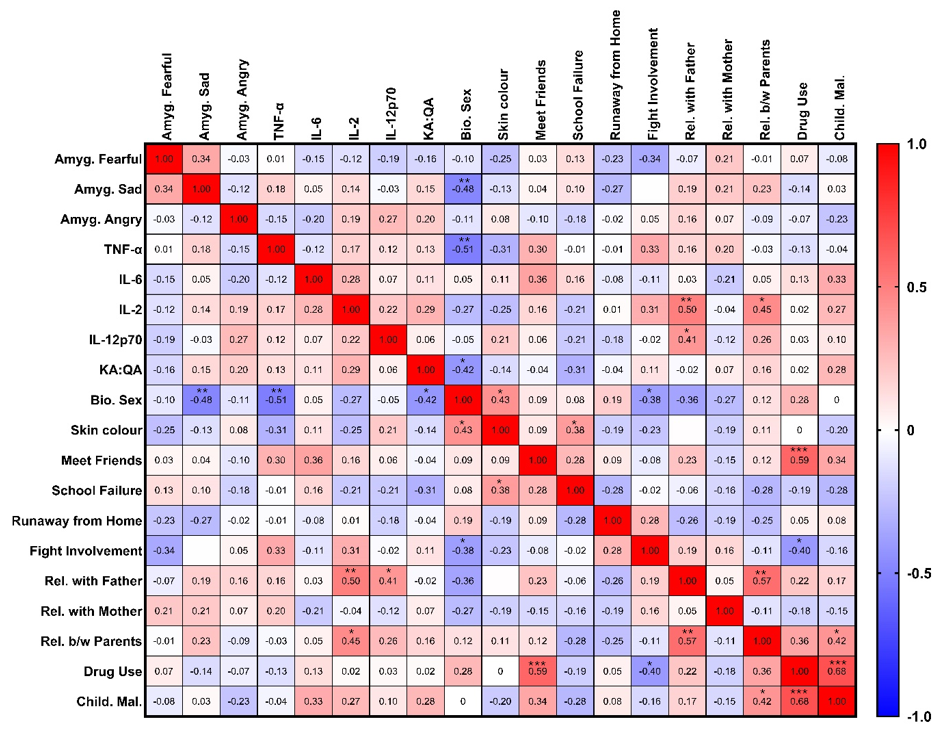


Negative correlation

Positive correlation

*

HH: high-risk IDEA-RS and high-risk IDEA-BIO-RS; Amyg.: amygdala; IL: interleukin; TNF-α: tumour necrosis factor alpha; KA:QA: kynurenic acid to quinolinic acid ratio; Bio. Sex: biological sex; Rel.: relationship; Rel. b/w: relationship between; Child.Mal.: childhood maltreatment
